# Mantonico and Pecorello Grape Seed Extracts: Chemical Characterization and Evaluation of *In Vitro* Wound-Healing and Anti-Inflammatory Activities

**DOI:** 10.3390/ph13050097

**Published:** 2020-05-14

**Authors:** Gabriele Carullo, Fabio Sciubba, Paolo Governa, Sarah Mazzotta, Luca Frattaruolo, Giorgio Grillo, Anna Rita Cappello, Giancarlo Cravotto, Maria Enrica Di Cocco, Francesca Aiello

**Affiliations:** 1 Department of Pharmacy, Health and Nutritional Sciences, Department of Excellence 2018-2022, University of Calabria, Edificio Polifunzionale, 87036 Rende (CS), Italy; gabriele.carullo@unical.it (G.C.); mazzottasarah04@gmail.com (S.M.); f.luca90@hotmail.it (L.F.); annarita.cappello@unical.it (A.R.C.); 2 Department of Chemistry, University of Rome “La Sapienza”, Piazzale Aldo Moro 5, 00185 Rome, Italy; Fabio.sciubba@uniroma1.it (F.S.); mariaenrica.dicocco@uniroma1.it (M.E.D.C.); 3 Department of Biotechnology, Chemistry and Pharmacy, Department of Excellence 2018-2022, University of Siena, Via Aldo Moro 2, 53100 Siena, Italy; paolo.governa@unisi.it; 4 Department of Organic and Medicinal Chemistry, Faculty of Pharmacy, University of Seville, C/O Prof. García González, 41071 Seville, Spain; 5 Department of Drug Science and Technology, University of Turin, Via P. Guria 9, 10125 Turin, Italy; giorgio.grillo@unito.it (G.G.); giancarlo.cravotto@unito.it (G.C.)

**Keywords:** Soxhlet, grape seeds, anti-inflammatory, wound healing, lipophilic fraction, carotenoids, HaCaT cell line, RAW 264.7 cell line, NMR, GC-MS

## Abstract

The winemaking process produces a huge number of pomaces that generally are used for energy purposes. Further valuable applications such as health-promoting properties are still under investigation. The seeds of the white berries of Mantonico and Pecorello *cv.* were extracted in a Soxhlet apparatus, using *n*-hexane and chloroform as solvents. Extracts were characterized by NMR and GC-MS analyses. They were assayed in vitro as wound healing and anti-inflammatory agents in HaCaT and RAW 264.7 cell lines, respectively. *n*-hexane Mantonico extract resulted in the most interesting wound healing sample, while *n*-hexane Pecorello, containing a good number of carotenoids, resulted in a good anti-inflammatory candidate. These preliminary findings underlined the benefit of grape seed extracts valorization due to their health-promoting properties.

## 1. Introduction

The recycling of by-products, especially those from plant-based materials, offers important advantages such as their ecologically friendly nature, low cost, availability, reduction of its storage, and elimination of the dismissal costs. Wine pomace (WP) consists of the solids remaining after the fermentation of grapes (*Vitis vinifera* L.). It includes solid grape parts such as skin, traces of pulp, and seeds with residues of stalk. WP revalorization is generally exploited to obtain antioxidants-enriched extracts, which could be combined with other ingredients in functional food matrixes [1,2,3]. Moreover, grape seed (GS) oil is an excellent cosmetic ingredient for controlling skin moisture, being easily absorbed by the skin [4]. Nowadays, several pharmaceutical preparations, containing GS-derived products, are available [5] and used for their health-promoting properties [6]. For instance, GS extracts (aqueous or alcoholic) have shown high antioxidant potential, leading to anti-atherosclerotic and anti-inflammatory effects [7], protection against cellular oxidative damage, modulation of antioxidant enzyme expression and protection against some types of cancer, in both human and animal models [8]. Among wine by-products, GSs are the most abundant and represent the portion of the fruit containing the higher concentration of bioactive molecules [9]. Furthermore, seeds showed the highest antioxidant activity, followed by skin and pulp. Indeed, GS have a great biological potential, which could be exploited by extracting bioactive compounds before addressing it as biomass for energy purposes. In this context, GSs can be considered a resource instead of waste [10]. In particular, the oils that can be extracted from seeds of different grape varieties own high value, as they contain large fractions of polyunsaturated fatty acids and they can be marketed at relatively high prices [11]. GS oil contains a large number of phenolic compounds, including flavonoids, carotenoids, phenolic acids, tannins, and stilbenes. This composition is tightly related to grapevine variety, environmental factors, and maturation degree of the grapes [8]. The healing of skin wounds and particularly chronic wounds is still a clinical emergency. Indeed, despite the many therapeutic tools that are available so far, none seems to be completely effective and safe, with the incomplete healing process leading to infection development, higher inflammatory state, reactive oxygen species (ROS) production and pain [12]. The topical application of a hydro-alcoholic GS extract has been reported to promote wound healing in rabbits [13]. Moreover, a proanthocyanidin-enriched GS extract, containing 5000 ppm resveratrol, was able to accelerate wound contraction and closure in mice, by upregulating VEGF expression in keratinocytes [14]. Other studies showed that GS oil could be an effective wound healing agent, thanks to the synergy between the antibacterial and antioxidant effect at the wound site. Furthermore, the topical application of a cream containing 2% GS extract led to the complete wounds closure in 8 days, which was faster than the placebo group (i.e., 14 days) [15]. Regarding the topical application, it was also shown that a GS extract embedded in hydrogel formulations was able to trigger wound healing, due to a synergistic action among hydrogel and bioactive components of the extract [16]. Interestingly, a GS oil, containing high amount of linoleic and oleic acid, was successfully used to increase the wound closure rate in rats [17]. The principal components of GS oil are generally fatty acids, well known to be useful remedies in wound healing disorders, especially associated to type-2 diabetes, employed alone or linked to polyphenols and other natural compounds [15,16,17,18]. The scope of this experimental work was to exploit the in vitro wound healing properties of four different fatty acid-enriched GS extracts, i.e., chloroform Mantonico (CM), chloroform Pecorello (CP), *n*-hexane Mantonico (HM), and *n*-hexane Pecorello (HP), obtained by Soxhlet extraction, derived from two autochthonous Calabrian cultivars, which, by far, have been only investigated for their vasoactive properties [5]. Furthermore, the anti-inflammatory potential, evaluated as the ability to attenuate the production of nitric oxide (NO) in RAW 264.7 macrophages cell line, was investigated for each extract, in order to assess if the extracts could have different therapeutic applications, that will be further analysed.

## 2. Results and Discussion

### 2.1. Extraction and Chemical Characterization of the Extracts

**Figure 1:**
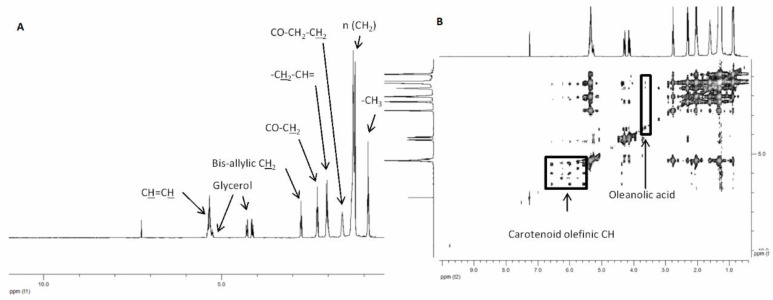
Representative ^1^H monodimensional (**A**) and ^1^H-^1^H TOCSY (**B**) NMR spectra of HP extract.

The procedure used to obtain the samples was a Soxhlet extraction with lipophilic solvents, able to extract apolar substances, preferentially. In particular, the extraction was conducted with chloroform and *n*-hexane because of their suitable affinity with fatty acids. Overall, the extracts were characterized through NMR and GC-MS analyses. The NMR technique was used to quantify the detected compounds in the extracts. In particular, the principal components recovered were fatty acids, including oleic and linoleic acids, present in good amounts in all the extracts. Appreciable amounts of high-quality products, oleanoic acid and carotenoids have been detected in all the samples, with HM containing the highest amount of fatty acids, glycerols, and phenolic compounds. HP contained the higher content of carotenoids, compared to the others (Table 1, Figure 1). All the NMR spectra are reported in Supporting Information (Figures S1–S8, Table S1).

The composition of the extracts was further examined via qualitative GC-MS analysis (Table 2), affording the volatile compounds of the samples. Different fatty acids, including saturated and non-saturated fatty acids, were recovered, including also different amines and aldehydes. All the GS-MS spectra are reported in Supporting Information (Figures S9–S12; Tables S2–S5).

### 2.2. Scratch Wound Healing Assay in HaCaT Cell Line

Table 3 shows the wound healing rate of untreated and TGF-β-stimulated HaCaT cells. The ability of the in vitro model to reproduce wound healing was confirmed by the fact that untreated cells were able to migrate toward the wound area after both 6 and 24 h. Moreover, TGF-β, used as a positive control, increased the wound healing rate after 24 h by approximately 20%, compared to the untreated control.

**Figure 2:**
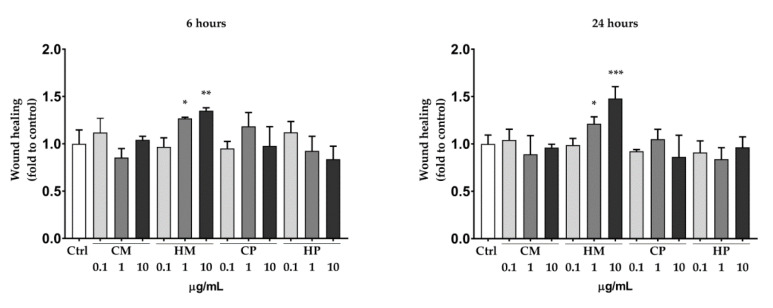
Scratch wound healing assay. The assay was performed 6 (**left**) and 24 (**right**) hours after treating HaCaT cells with increasing concentrations (0.1 to 10 μg/mL) of different extract (as indicated) or DMSO (Ctrl). Data are expressed as mean ± SD of three independent experiments and normalized to the Ctrl value * *p* < 0.05 vs. control; ** *p* < 0.01 vs. control; *** *p* < 0.001 vs. control by one-way ANOVA.

The concentrations of the sample for the biological assays were chosen on the basis of cell viability experiments (Figure S13, Supporting information). CM, CP, and HP did not stimulate the wound healing rate, compared to the untreated control. Notably, HM was able to increase the keratinocytes wound healing in a concentration-dependent manner after 6 and 24 h (Figure 2).

**Figure 3:**
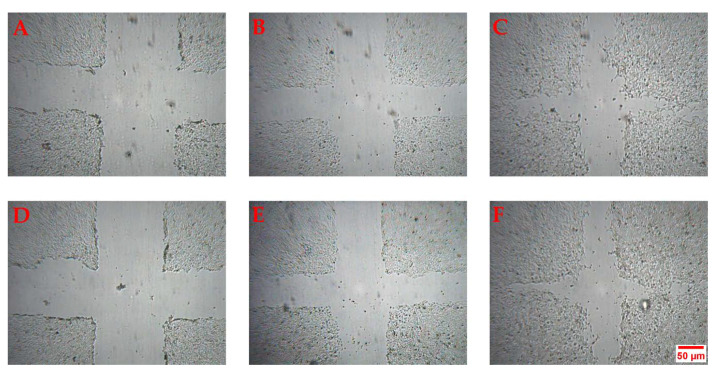
Representative images of the scratch wound healing assay. The images show the effect of Ctrl at t0 (**A**), 6 (**B**) and 24 h (**C**) and HM 10 µg/mL at t_0_ (**D**), 6 (**E**) and 24 h (**F**).

The maximum effect was obtained with the concentration of 10 µg/mL after 24 h (+50% compared to the untreated control). Representative images showing the effect of HM 10 µg/mL on HaCaT cells wound healing are reported in Figure 3. These results are consistent with the chemical composition of the extracts, with HM containing the higher amount of fatty acids, compared to the others. Indeed, fatty acids have been reported to induce beneficial effect on different phases of the wound healing process [18,19,20]. Moreover, we recently reported that hybridization of quercetin and oleic acid led to increased keratinocytes wound healing by targeting the FFA1/GPR40. Compared to quercetin alone, the hybrid demonstrated a better biological effect, highlighting the importance of fatty acid moiety [21,22].

**Figure 4:**
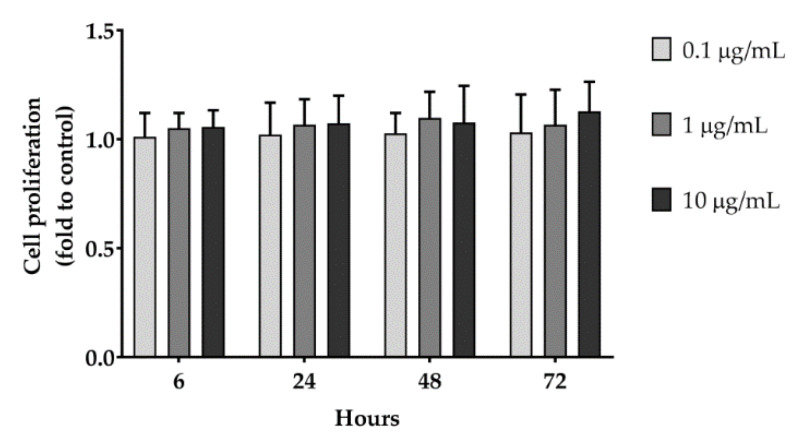
Cell proliferation assay. HaCaT cells were treated with increasing concentrations (0.1 to 10 μg/mL) of different extract (as indicated) or DMSO (Ctrl). The SRB absorbance at 540 nm was recorded 6, 24, 48, and 72 h after treatment. Data are expressed as mean ± SD of three independent experiments and normalized to the Ctrl value. One-way ANOVA was used for determining the statistical differences between groups.

The wound healing effect of HM seems to be related to the stimulation of keratinocytes migration, as no increase of cell proliferation were observed in the SRB assay after 6, 24, 48, and 72 h (Figure 4). The migration-specific effect could be beneficial, as abnormal keratinocytes proliferation can alterate the epidermal mesenchymal interactions, leading to delayed reepithelialzation or hypertrophic scars [23].

**Figure 5:**
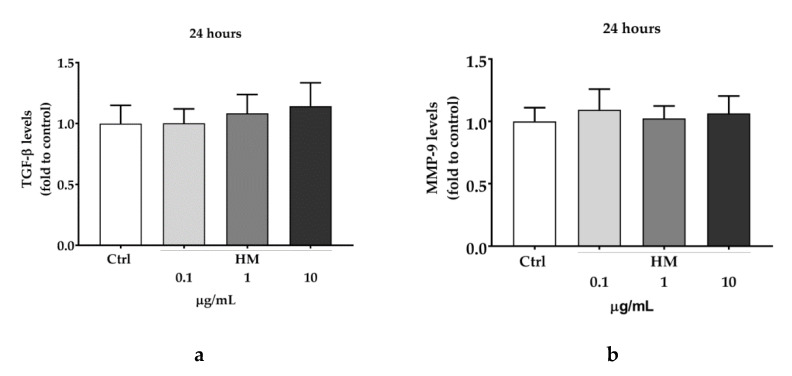
Effect of HM on TGF-β levels (**a**) and MMP-9 release (**b**). HaCaT cells were treated with increasing concentrations (0.1 to 10 μg/mL) of different extract (as indicated) or DMSO (Ctrl) for 24 h. TGF-β levels and MMP-9 release were assessed by non-competitive sandwich ELISA. Data are expressed as mean ± SD of three independent experiments and normalized to the Ctrl value. One-way ANOVA was used for determining the statistical differences between groups.

In the attempt to better understand the mechanism of action of HM, we dosed the intracellular levels of TGF-β and the release of MMP-9. However, as reported in Figure 5, no significant difference was observed between the HM-treated cells and the untreated control, even if a tendency to increase TGF-β could be noted with the higher concentration of HM.

### 2.3. Inhibition of Nitric Oxide Production in Lipopolysaccharide (LPS)-Stimulated RAW 264.7 Macrophages

**Figure 6:**
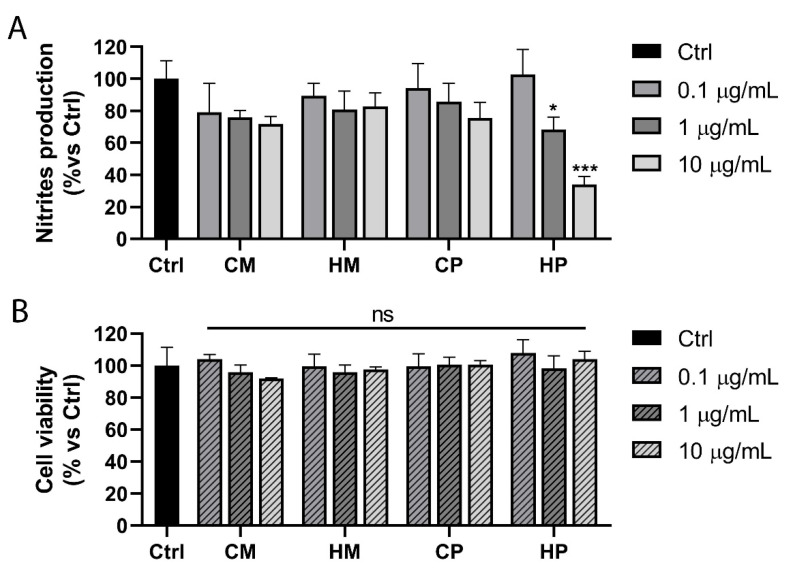
Anti-inflammatory potential of the extracts. (**A**) Nitrites production assessment, by Griess assay, in lipopolysaccharide (LPS)-stimulated RAW 264.7 cells treated with increasing doses (0.1 to 10 μg/mL) of different extract (as indicated) or DMSO (Ctrl). Data are expressed as percentage of nitrite production *versus* control (DMSO-treated cells). (**B**) Viability assessment, by MTT assay, of cells treated as indicated above. Values represent mean ± SD of three independent experiments. * *p* < 0.05; *** *p* < 0.001; ns = not significant.

The anti-inflammatory potential of the extracts was assessed by monitoring their ability to inhibit nitric oxide (NO) production in lipopolysaccharide (LPS)-stimulated RAW 264.7 murine macrophages, which are widely used for preliminary in vitro studies of anti-inflammatory candidates. Indeed, through the TLR4 receptor, the bacterial lipopolysaccharide (LPS) is able to activate the inflammation. The successive activation of the transcription factor Nuclear Factor Kappa B (NF-κB) stimulates the expression of inflammatory related enzymes [24]. Among these, the inducible nitroxide synthase (iNOS) is responsible for the NO synthesis following the inflammatory stimulus. Our results, shown in Figure 6, revealed a marked dose-dependent anti-inflammatory activity for the HP extract, while no significant effect was observed with the other extracts, at the tested concentrations. Moreover, the analysis of cell viability, by using MTT assay, demonstrated the absence of toxic effects mediated by the extracts, confirming that the NO reduction observed for HP extract is actually the result of interactions with the inflammatory pathway and not the reflection of a reduced cell viability. By weaving the results obtained with the composition of the different extracts, it emerges that the highest carotenoid content may underlie the HP anti-inflammatory potential. Indeed, with the same extraction method, Pecorello grapevine cultivar proved to be richer in carotenoids than Mantonico cultivar. Carotenoids are natural products with known antioxidant and anti-inflammatory power [25,26], mediated both by the activation of the transcription factor Nrf2, responsible for the cellular anti-inflammatory response, and by the inactivation of the NF-κB, which mediates pro-inflammatory effects instead. The inhibition of NO production by HP, observed in our experimental conditions, could therefore be the result of inhibitory effects of carotenoids on the NF-κB pathway.

## 3. Materials and Methods

### 3.1. Plant Material

Pomaces derived from Mantonico (M) and Pecorello (M) white grapes were provided from Le Moire srl (ctr Strivillati Motta Santa Lucia, Catanzaro, Italy) of Dr. Paolo Chirillo (Latitude: 39°05′28″ N–Longitude: 16°17′35″ E–Altitude: 527 m) and harvested in September and October 2018. All the samples were stored at −18 °C until use.

### 3.2. Chemicals

All the solvents used, including *n*-hexane, chloroform and methanol were of ultrapure grade, used without further purification and acquired from Levanchimica s.r.l. (Bari, Italy). Chloroform-*d* (CDCl_3_) and 3-(Trimethylsilyl)propionic-2,2,3,3-d4 acid sodium salt (TSP) were bought from Merck (Darmstadt, Germany).

### 3.3. Extraction Procedure

Seeds were separated from the skins and milled to obtain a fine powder using a blender type A11 basic. The powder was then freeze-dried and stored at −18 °C until use. Two Soxhlet extractions were performed starting from 15 g of powders employing 500 mL of *n*-hexane (H) and chloroform (C), respectively, for 8 h. The solvent was evaporated at 40 °C using a Buchi rotavapor R-II (Postfach 9230 Flawil, Switzerland) to give four different extracts: HM (3.20 g), HP (2.95 g), CM (2.31 g), CP (2.35 g).

### 3.4. Gas-Chromatography/Mass Spectrometry (GC/MS) Analysis

The composition of the extracts CM, CP, HM, and HP was determined by GC-MS qualitative analyses, performed in an Agilent Technologies 6850 Network GC System, using a 5973 Network Mass Selective Detector, a 7683B Automatic Sampler (Santa Clara, CA, USA), and a capillary column (HP-5MS 5% Phenyl Methyl Siloxane, length 30 m, i.d. 0.25 mm, film thickness 0.25 μm). All the lipophilic extracts (~10 mg) were dissolved in 1 mL of *n*-hexane and peak identification was made by means of Wiley7n and NIST11 GC libraries. All the reported compounds have more than 80% of overlap with the references. Relative integrations were made for sake of comparison between the analysed samples.

### 3.5. Nuclear Magnetic Resonance (NMR) Analysis

Extracts were analysed with ^1^H NMR, ^1^H-^1^H TOCSY homonuclear bidimensional experiments in order to establish, when possible, the quantitative amount of substances. Samples (1 mg) were re-suspended in 600 μL of CDCl_3_ containing TSP (2 mM) as internal standard. Mono-dimensional ^1^H NMR spectra were acquired after 64 scans for each spectrum on a Bruker Avance 400 spectrometer (Bruker Spectrospin, Karlsruhe, Germany) operating at 9.4 T at 298 K using a 90° detection pulse and acquiring the FIDs in 64 K points, the spectral width was 6009.15 Hz. The solvent signal was suppressed by means of a presaturation scheme and the relaxation delay was set to 6.55 s in order to achieve a 15 s total acquisition time to avoid relaxation effects. Homonuclear ^1^H-^1^H TOCSY experiments were performed in order to ensure signal assignment. TOCSY experiments were acquired with a spectral width of 6009.15 Hz in both dimensions, a data matrix of 8 K × 256 points, a mixing time of 110 ms and relaxation delay of 2 s. Monodimensional ^1^H spectra were analyzed with ^1^D- NMR Manager software ver. 12 (ACD/Labs, Toronto, ON, Canada): FIDs underwent exponential multiplication (LB = 0.09 Hz), were Fourier transformed, and phase and baseline corrected. Bidimensional spectra were processed with Bruker Topspin ver. 2.1. The assignment of the resonances was performed by analysing 1H NMR characteristics and cross-correlated signals in 2D spectra and by comparison with the literature compilations. Quantification of the identified compounds was performed by comparison of the signal integral with the reference one, and quantities were expressed in mg of compound normalized for the aliquot weight expressed in g.

### 3.6. Cell Cultures and Treatments

Human keratinocytes from adult skin (HaCaT) were cultured in 75 cm^2^ flasks (Sarstedt, Milan, Italy) in complete Dulbecco’s Modified Eagle’s medium (DMEM), which was composed of DMEM (Sigma-Aldrich, Milan, Italy) medium with 10% heat-inactivated fetal bovine serum (FBS, Sigma-Aldrich) and 1% L-glutamine (Sigma-Aldrich). Murine macrophages RAW 264.7 cell line was obtained from the American Culture Collection (ATCC, Manassas, VA) and cultured in DMEM supplemented with 10% FBS, 2 mM L-glutamine and 1% penicillin/streptomycin (Gibco, Life Technologies, Waltham, MA, USA). All cells were cultured at 37 °C in a humidified atmosphere containing 5% CO_2_. Number of cell passages was kept under 20 from the reference culture (ATCC) [27]. EDTA-trypsin (Sigma-Aldrich) solution was used for detaching cells from flasks, and cell counting was performed using a hemocytometer by Trypan Blue staining. Samples were solubilized in dimethyl sulfoxide (DMSO) and diluted in complete medium in order to reach the final concentrations. DMSO concentration in each final treatment was maintained below 0.1% and the untreated control was represented by the same amount of DMSO present in the treatments.

### 3.7. HaCaT Cell Viability Assay

Cell viability was assessed using the cell counting kit 8 (CCK-8, Sigma-Aldrich) as previously described [28]. Briefly, 5 × 10^4^ cells/well were seeded into 96-well plates and grown to confluence. Medium was then replaced with 100 µL of fresh DMEM containing increasing concentrations of the samples to the appropriate well. After the incubation time, 10 µL of CCK-8 was added to each well and incubated for 1 h. Absorbance was measured at 450 nm using a MP96 microplate reader spectrophotometer (Safas, Monte Carlo, Principality of Monaco). Treatments were performed in sextuplicate in three independent experiments and cell viability was calculated by normalizing the absorbance of the test wells to the untreated control.

### 3.8. HaCaT Scratch Wound Healing Assay

The scratch wound-healing assay was applied by slightly modifying the protocol of Chen (2012) as previously described [12,21,29]. Briefly, HaCaT cells (5 × 10^4^) were seeded into six-well cell culture plates and allowed to grow to 70–80% confluence as a monolayer. The monolayer was gently scratched across the center of the well with a sterile one-mL pipette tip. A second scratch was performed in a perpendicular way to the first, creating a cross in each well. After scratching, the medium was removed, and the wells were washed twice in PBS (Sigma-Aldrich) solution. Fresh medium containing 5% *v/v* of heat-inactivated FBS and treatments was added to each well, and cells were grown for 24 h. Images were obtained from the same fields immediately after scratching (t_0_) and after six and 24 h using a Leica DMIL microscope and were analyzed using ImageJ software by manually selecting the wound region and recording the total area.

The experiments were conducted in duplicate, in three independent experiments. Untreated scratched cells represented the control.

The percentage of wound closure was calculated using the following formula:

% Wound closure = [(Wound area t_0_ − Wound area t)/Wound area t_0_] × 100

where t is the time in hours.

Results was then normalized to the control group.

### 3.9. HaCaT Cell Proliferation Assay

Cell proliferation was assessed by means of sulforhodamine B (SRB, Sigma-Aldrich) assay, as previously reported [21]. Briefly, HaCaT cells (1 × 10^4^) were seeded into 96-well plates and allowed to grow for 24 h. Cells were then treated with increasing concentrations of HM and incubated for 6, 24, 48 and 72 h. After the incubation time, medium were replaced and cells were fixed by adding trichloroacetic acid to a final concentration of 10% (v/v) for one h at 4 °C. Cells were washed, and 0.4% SRB solution was added to each well and incubated for 30 min at rt. Cells were then washed with 1% (v/v) acetic acid, and SRB was solubilized with 10 mM of Tris (Sigma-Aldrich, pH = 10.5). Absorbance was recorded at 540 nm using a MP96 spectrophotometer (Safas, Monaco). Treatments were performed in sextuplicate in three independent experiments, and cell proliferation was calculated by normalizing the absorbance of the test wells to the untreated control.

### 3.10. TGF-β Production and MMP-9 Release

After the scratch wound-healing assay, supernatants were collected, centrifuged (4 °C, 15,000 *g* × 15 min), and frozen at −80 °C for MMP-9 dosage. Adherent cells were washed twice in PBS and lysed for TGF-β dosage. Protein content of sample lysate was determined by Bradford protein assay [30]. TGF-β production and MMP-9 release were assessed by non-competitive “sandwich” ELISA kit (Biolegend e-Bioscience DX Diagnostic, Monza, Italy), according to the provider datasheet. Absorbance was recorded at 450 nm using a SAFAS MP96 spectrophotometer.

### 3.11. Inhibition of Nitric Oxide (NO) Production in Lipopolysaccharide (LPS)-Stimulated RAW 264.7 Cells

Inhibition of NO production was assessed as previously reported [31]. Briefly, RAW 264.7 cells were seeded in 24 well plates, at a density of 2 × 10^5^ cells/well and incubated overnight in complete medium. The following day, medium was replaced with fresh DMEM and then cells were treated for 24 h with LPS (1 µg/mL) and different concentrations of extracts (ranging from 0.1 to 10 µg/mL). DMSO was used as a vehicle control. After 24 h, 100 µL of cell culture supernatant were incubated with 100 µL of Griess reagent (Sigma-Aldrich) at room temperature for 10 min in a 96-well plate and then, to quantify nitrites, stable products of NO, the absorbance was measured at 550 nm using a microplate reader (Synergy H1 microplate reader, BioTek, Winooski, USA). The absence of treatment-induced cytotoxic effects on RAW 264.7 cells was verified monitoring cell viability by MTT assay, as previously described [32]. LPS-stimulated RAW 264.7 cells were treated with increasing doses (0.1 to 10 μg/mL) of the extracts (as indicated) or DMSO (Ctrl). Data were expressed as percentage of nitrite production versus control (DMSO-treated cells).

### 3.12. Statistical Analysis

The statistical differences were determined by the analysis of the variance (ANOVA). Shapiro-Wilk normality test was used to check the normality distribution of variables. Values are expressed in the range of +/− standard deviation and *p* < 0.05 was considered statistically significant. Graphs and calculations were performed using Graphpad Prism.

## 4. Conclusions

Wine-making process furnishes large amounts of pomace that could be exploited for their health-promoting properties. The white berry pomaces are still scarcely investigated, furnishing the opportunity to develop new valuable pharmaceutical ingredients. In this work, we compared the GS extracts of Mantonico and Pecorello, and the differences of fatty acids profiles and other phytochemicals, such as carotenoids and polyphenols. In vitro, the linoleic acid-rich extract HM resulted a useful wound healing agent, while HP, rich in carotenoids, showed the most suitable anti-inflammatory activity, opening the way for translational or technological investigations of agro-food by-products.

## Figures and Tables

**Table 1:** ^1^H-NMR quantitative analyses of extracts.

Compounds ^1^	Assignment ^2^	Multiplicity ^3^	δ H (ppm)	Amount (µmol/g) ^4^			
				CM	CP	HM	HP
Stearic acid	CH_2_-CO_2_^-^	t	2.30	24.71	14.21	269.58	69.87
Oleic acid	CH_2_-CH=CH	m	2.03	33.93	18.47	375.04	97.44
Linoleic acid	=CH-CH_2_-CH=	t	2.76	92.71	64.04	1177.66	277.85
Oleanoic acid	CH-3	m	3.60	3.24	1.79	17.79	4.21
Glycerol	CH_2_	dd	3.65–3.55	48.57	31.31	587.87	142.58
Carotenoids	CH-11,11′	m	6.68	0.77	0.54	2.69	7.19
Total Phenols	Aromatic moieties	m	6.8–7.0	3.11	1.46	3.02	0.00
Aldehydes	CHO	brs	9.76	0.35	0.09	2.90	0.87

^1^ Metabolites identified in the ^1^H NMR spectrum of the (C) and (H) extracts of seeds. ^2^ chosen for metabolite quantification. ^3^ s: singlet, brs: broad singlet, t: triplet, dd: doublet of doublets, m: multiplet. ^4^ The SD associated to the measure is 5% of the value.

**Table 2:** GC-MS analysis of the extracts.

	CM	CP	HM	HP
Compounds	% of compound			
1,2-Benzenedicarboxylic acid, bis(2-methylpropyl) ester	-	-	2.19	0.53
2,4 Decadienal	13.43	-	9.76	10.37
2,4-Decadienal, (*E*,*E*)-	9.17	15.59	-	5.83
2-Decenal, (*E*)-	-	5.01	-	1.48
2-Decenal, (*Z*)-	-	-	4.02	-
*E*-2-dodecenal	-	-	-	0.93
Eicosane	-	-	5.57	0.48
2-Heptenal, (*E*)-	12.45	12.59	12.45	4.53
13-Hexacosyne	-	-	2.41	-
Hexadecanoic acid	5.10	5.58	3.96	8.03
Hexadecanoic acid, ethyl ester	-	-	-	0.89
Hexane, 1,1-diethoxy-	-	1.58	5.27	1.18
Linoleic acid, butyl ester	2.91	-	-	-
Linoleic acid, ethyl ester	-	9.87	-	-
9,12-Octadecadienoic acid, ethyl ester	-	-	-	12.30
9,12-Octadecadienoic acid (*Z*,*Z*)-	42.50	31.19	6.59	49.60
Octadecane	-	-	2.53	-
Octadecanoic acid	11.22	-	7.58	-
9-Octadecenoic acid (*Z*)-	-	-	14.09	-
1-Octanamine, *N*-methyl-*N*-octyl-	-	-	3.31	-
1-Octanamine, *N*,*N*-dioctyl-	-	-	2.13	-
2 Octenal	-	1.58	-	-
2-Octenal, (*E*)-	-	-	-	0.59
1-Octen, 3-ol	-	2.09	-	-
Phthalic acid, isobutyl nonyl ester	-	0.51	-	-
Tetradecanoic acid, ethyl ester	-	0.98	-	-

-: not detected.

**Table 3:** Wound healing percentage of untreated and TGF-β-stimulated HaCaT cells.

Treatment	6 h	24 h
Ctrl	19.39 ± 3.18	36.64 ± 3.86
TGF-β ^a^	17.06 ± 1.72	43.87 ± 2.58

^a^ 4 ng/mL.

## Supplementary Materials

The following are available online at https://www.mdpi.com/1424-8247/13/5/97/s1, Figures S1–S8: Mono- and Bi-dimensional spectra of the extracts. Table S1. Table of resonance assignments; Figures S9–S12: GC-MS qualitative analysis for the extracts; Table S2–S5: Identification of peaks in the extracts. Figure S13. HaCaT cell viability assay.
