# Supplementary material for: Mantonico and Pecorello Grape Seed Extracts: Chemical Characterization and Evaluation of In Vitro Wound-Healing and Anti-Inflammatory Activities
Source: Pharmaceuticals (Basel). 2020 May 14;13(5):97. doi: 10.3390/ph13050097 (PMC7281466; doi:10.3390/ph13050097)
Supplement: Supplementary file 1 [file pharmaceuticals-13-00097-s001.pdf]

SUPPORTING INFORMATION

Mantonico and Pecorello grape seed extracts: chemical characterization and evaluation of *in vitro* wound-healing and anti-inflammatory activities

Gabriele Carullo[1], Fabio Sciubba[2], Paolo Governa[3], Sarah Mazzotta[1,4], Luca Frattaruolo[1], Giorgio Grillo[5], Anna Rita Cappello[1], Giancarlo Cravotto[5], Maria Enrica Di Cocco[2] and Francesca Aiello[1,*]

[1] Department of Pharmacy, Health and Nutritional Sciences, University of Calabria, Edificio Polifunzionale, 87036, Rende (CS), Italy.
[2] Department of Chemistry, University "La Sapienza" Rome, Piazzale Aldo Moro 5, 00185, Rome, Italy.
[3] Department of Biotechnologies, Chemistry and Pharmacy, University of Siena, Via Aldo Moro 2, 53100, Siena, Italy.
[4] Department of Organic and Medicinal Chemistry, Faculty of Pharmacy, University of Seville, C/O Prof. Garcia Gonzalez, 41071, Seville, Spain.
[5] Department of Drug Science and Technology, University of Turin, Via P. Giuria 9, 10125, Turin, Italy.
* Correspondence: francesca.aiello@unical.it ; Tel.: +39 0984 493154

INDEX

Figure S1. Monodimensional 1H spectrum of CM
Figure S2. Bidimensional ^{1}H-^{1}H TOCSY spectrum of CM
Figure S3. Bidimensional ^{1}H-^{13}C HSQC spectrum of CM
Figure S4. Monodimensional ^{1}H spectrum of CP
Figure S5. Monodimensional ^{1}H spectrum of HM
Figure S6. Monodimensional ^{1}H spectrum of HP
Figure S7. Bidimensional ^{1}H-^{1}H TOCSY spectrum of HP
Figure S8. Bidimensional ^{1}H-^{13}C HSQC spectrum of HP
Table S1. Table of resonance assignments
Figure S9. GC-MS qualitative analysis for CM extract
Table S2. Identification of CM peaks
Figure S10. GC-MS qualitative analysis for CP extract
Table S3. Identification of CP peaks
Figure S11. GC-MS qualitative analysis for HM extract
Table S4. Identification of HM peaks
Figure S12. GC-MS qualitative analyses for HP extract
Table S5. Identification of HP peaks
Figure S13. HaCaT cell viability assay

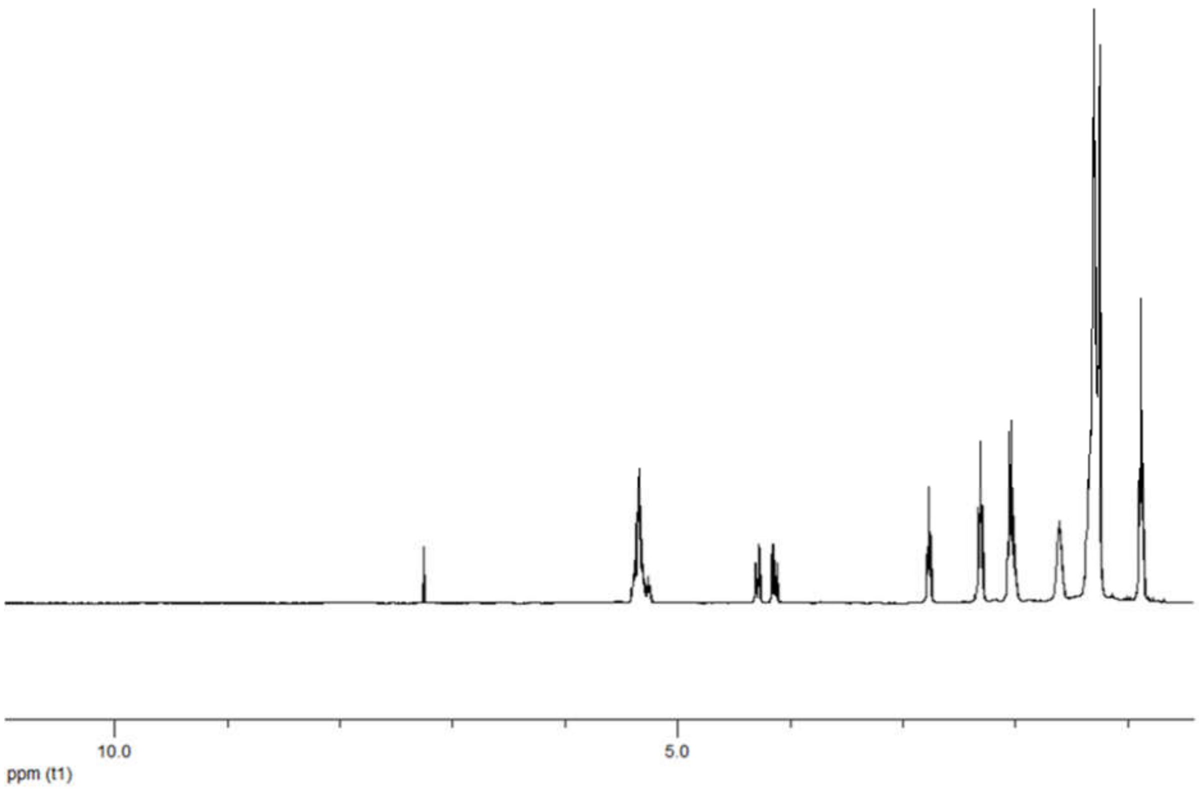

Figure S1. Monodimensional ^{1}H spectrum of CM

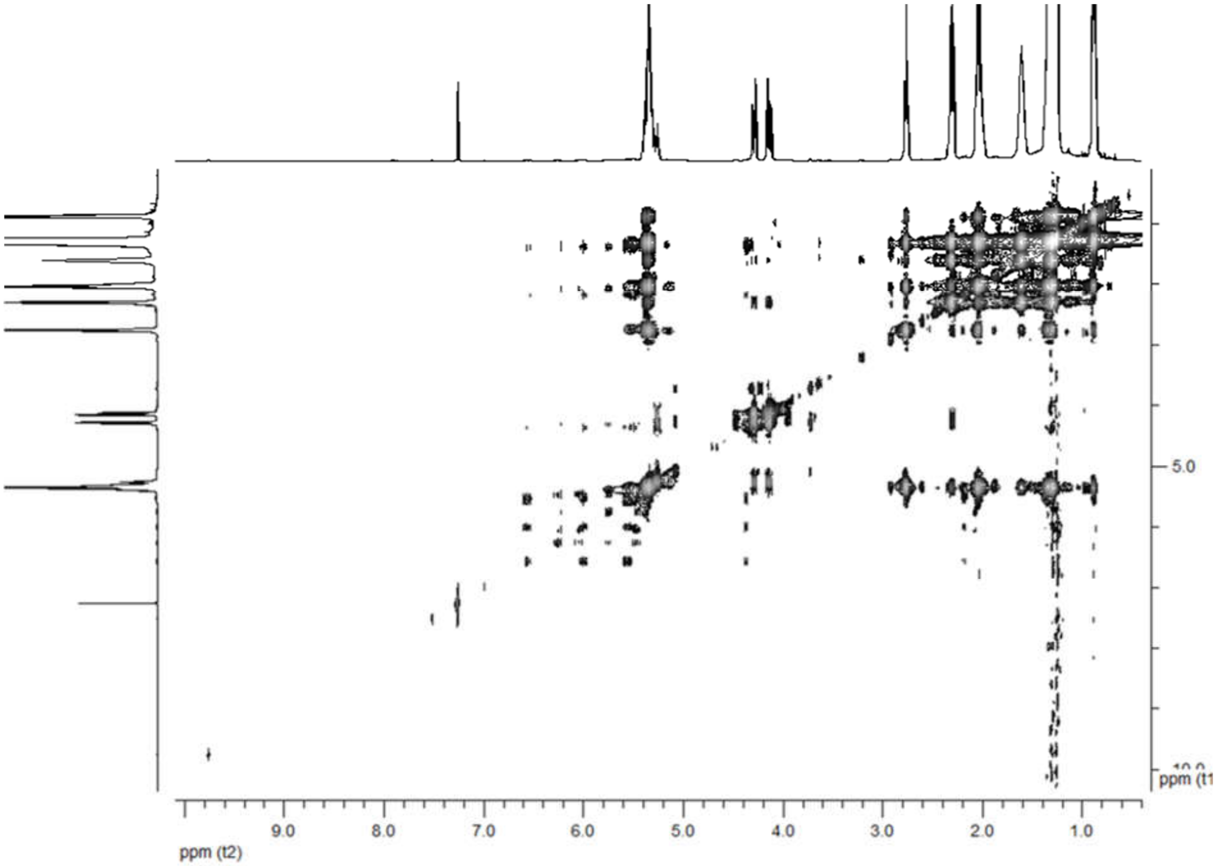

Figure S2. Bidimensional ^{1}H-^{1}H TOCSY spectrum of CM

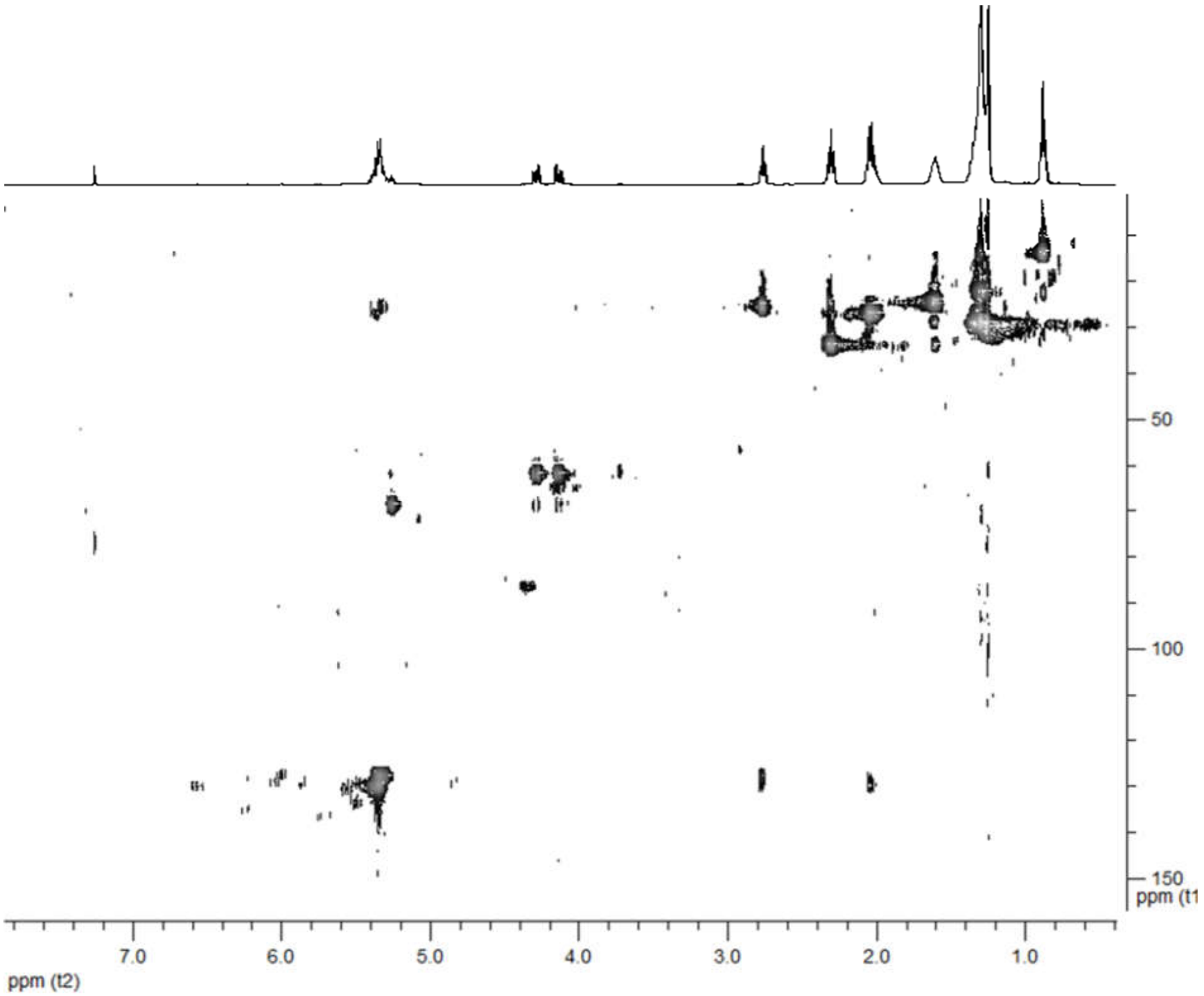

Figure S3. Bidimensional ^{1}H-^{13}C HSQC spectrum of CM

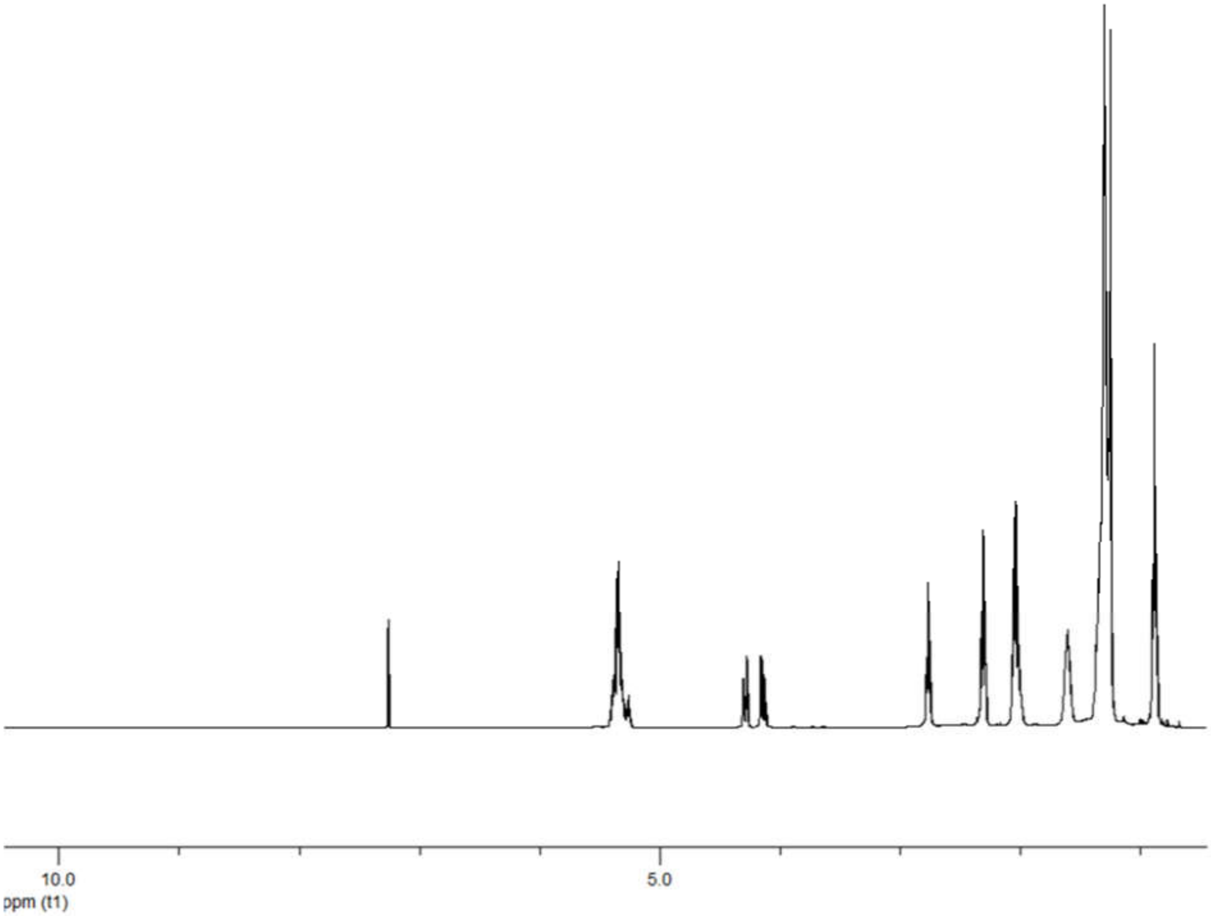

Figure S4. Monodimensional ^{1}H spectrum of CP

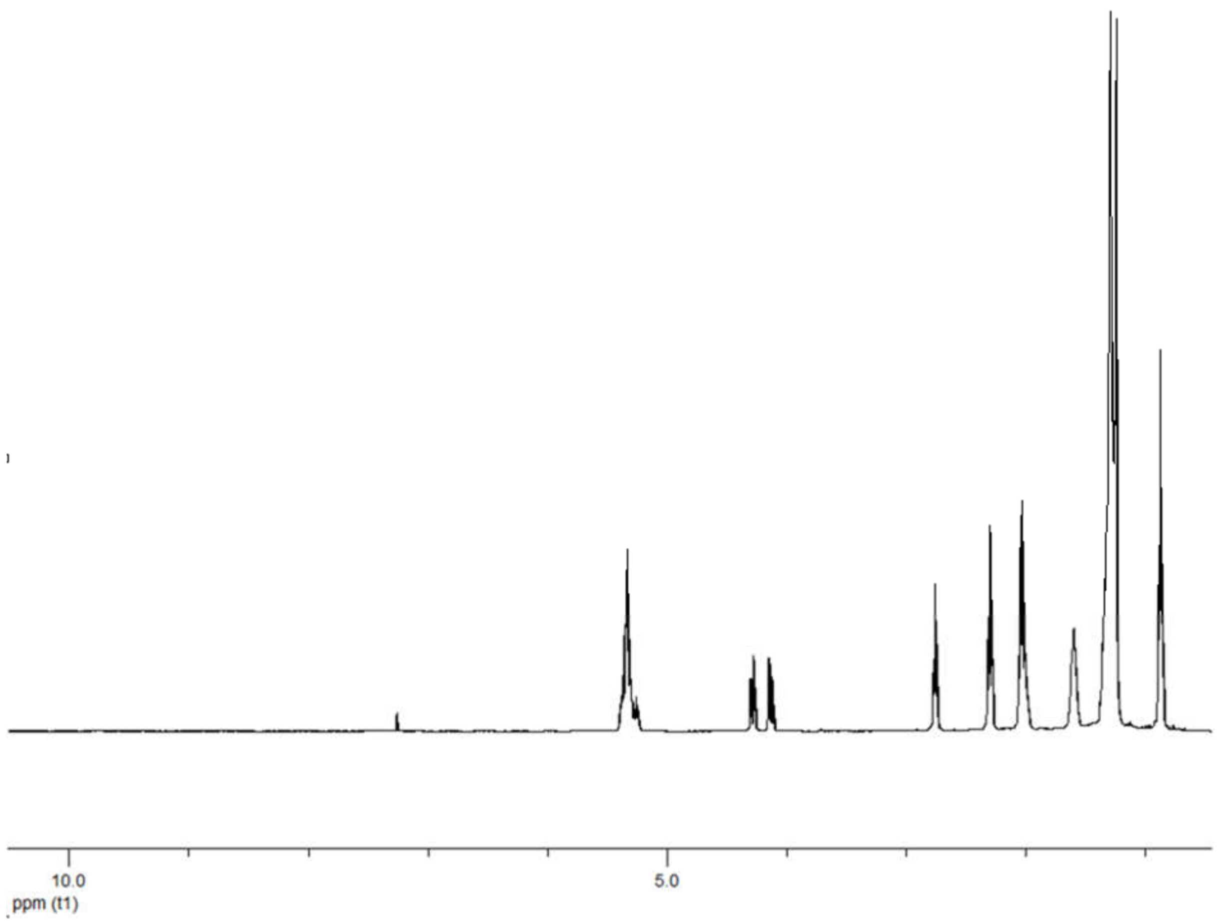

Figure S5. Monodimensional ^{1}H spectrum of HM

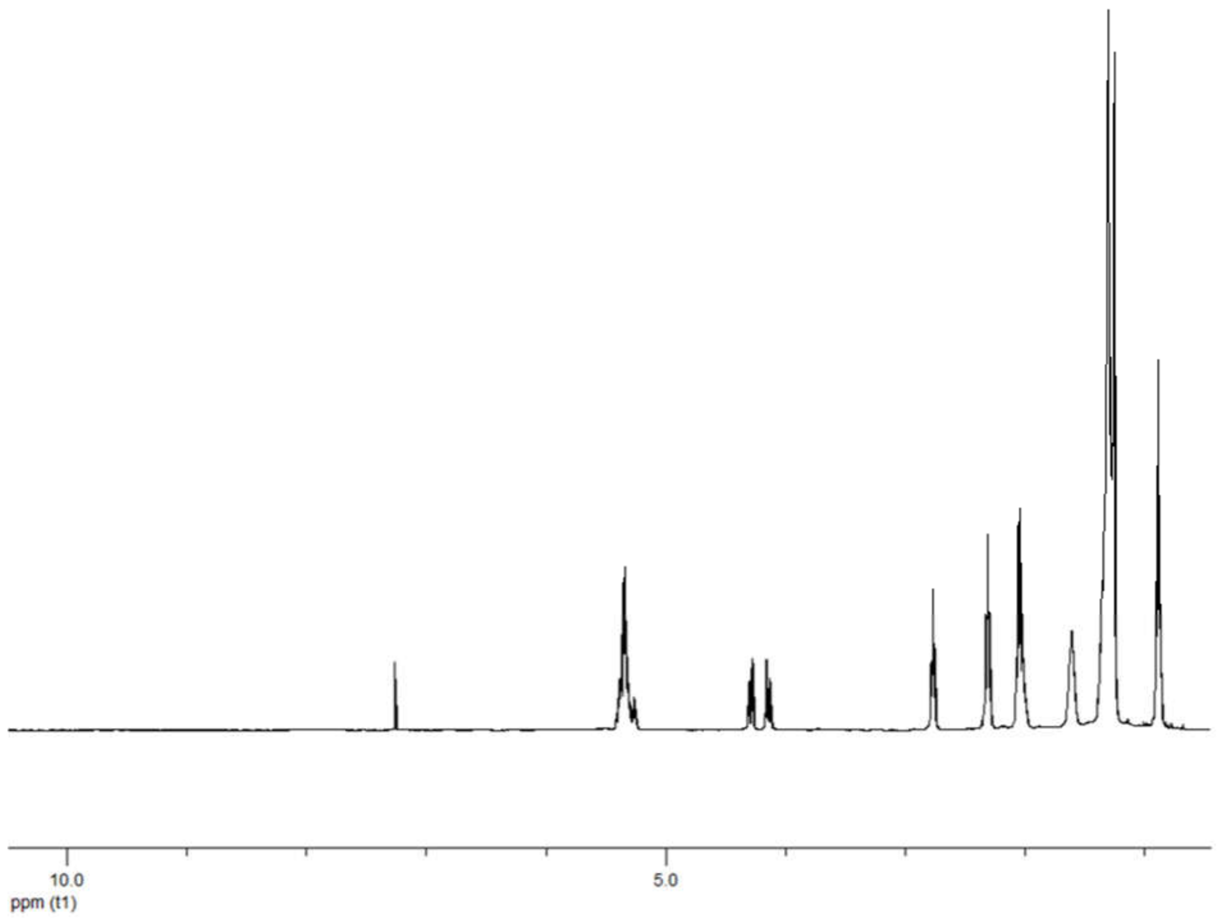

Figure S6. Monodimensional ^{1}H spectrum of HP

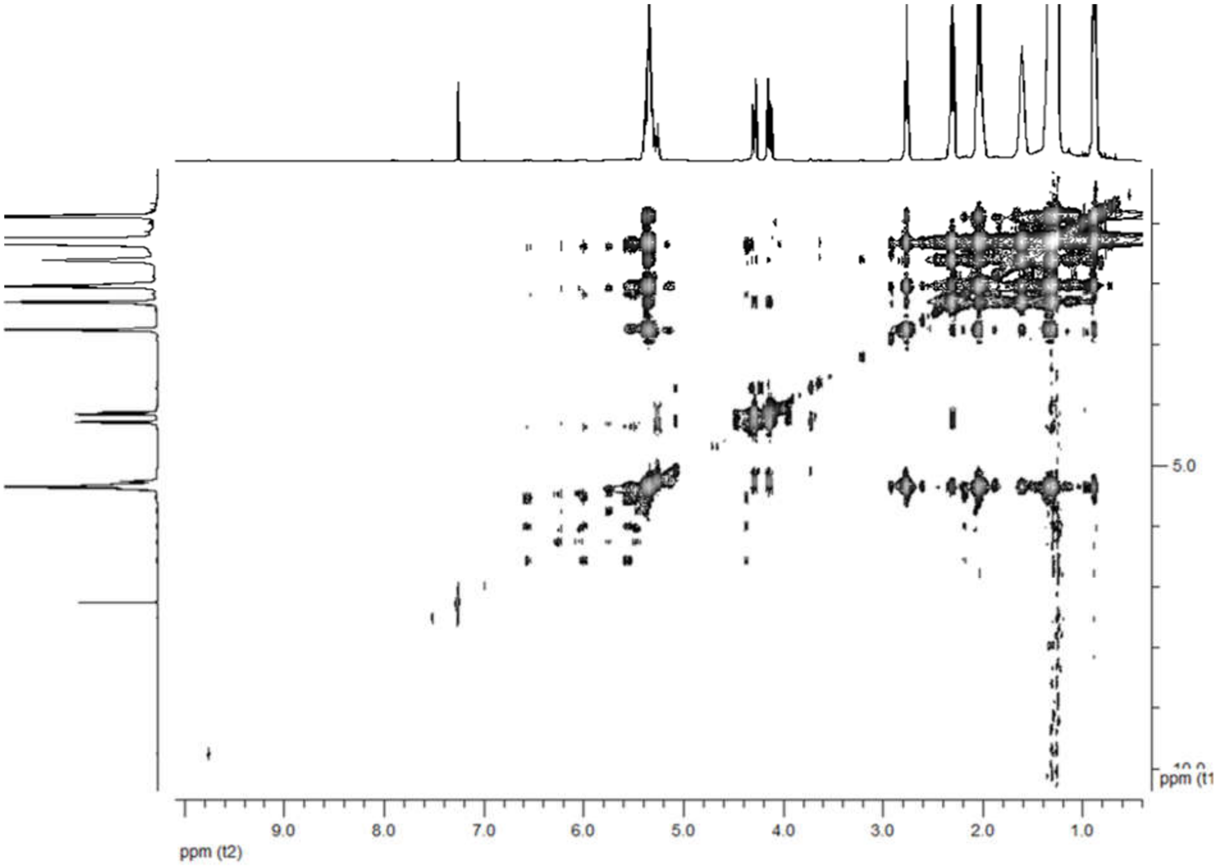

Figure S7. Bidimensional ^{1}H-^{1}H TOCSY spectrum of HP

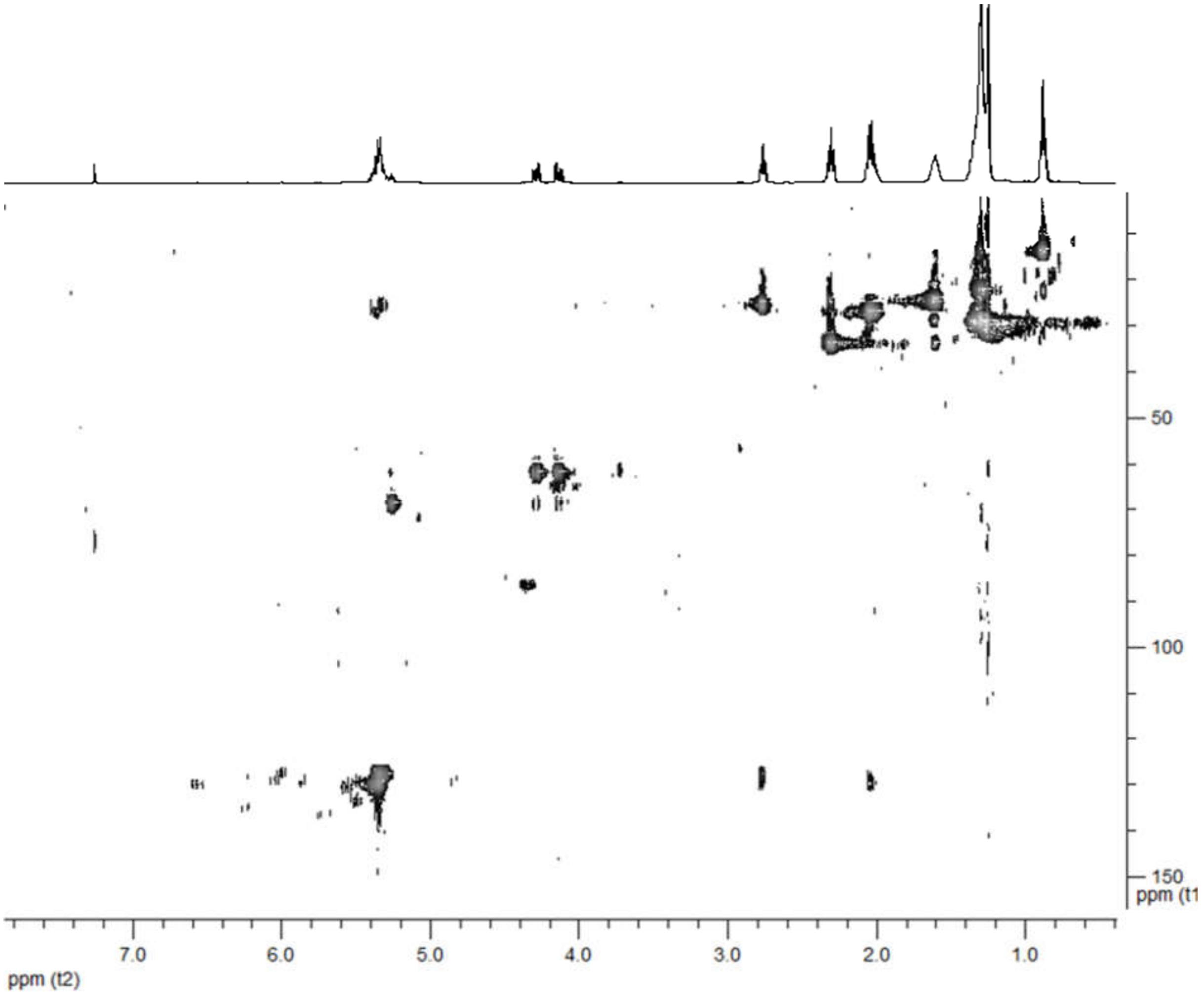

Figure S8. Bidimensional ^{1}H-^{13}C HSQC spectrum of HP

Table S1. Table of resonance assignments

**Compound** [1]	**Assignment** [2]	**^{1}H δ (ppm)**	**Multiplicity** [3]	**^{13}C δ (ppm)**
**Stearic acid**	CH_3	0.87	t	14.05
	n-CH_2	1.26	m	29.32
	$C\underline{H}_2$-CH_2-CO_2^-	1.62	m	24.61
	**CH_2-CO_2^-**	**2.30**	**t**	33.52
**Oleic acid**	CH_3	0.88	t	14.07
	n-CH_2	1.27	m	29.35
	**CH_2-CH=CH**	**2.03**	**m**	27.14
	CH=CH	5.35	m	129.84; 127.43
	$C\underline{H}_2$-CH_2-CO_2^-	1.62	m	24.61
	CH_2-CO_2^-	2.31	t	33.96
**Linoleic acid**	CH_3	0.86	t	14.06
	n-CH_2	1.36	m	29.37
	$C\underline{H}_2$-CH=CH	2.04	m	29.45
	CH=CH	5.37	m	130.29; 128.45
	**=CH-$C\underline{H}_2$-CH=**	**2.76**	**t**	25.68
	$C\underline{H}_2$-CH_2-CO_2^-	2.06	m	24.75
	CH_2-CO_2^-	2.31	t	34.05
**Monoacylglycerol**	**CH_2**	**3.65-3,55**	**dd**	65.45
	CH_2	4.05-4.15	dd	70.32
	CH	3.82	m	75.12
**Triglyceride**	CH	5.13-5.21	m	77.45
	**2CH_2**	4.15-**4.29**	**dd**	68.23
**Oleanoic acid**	CH2-1	0.85	t	45.4
	CH2-2	1.44	m	24.5
	**CH-3**	**3.60**	**m**	**76.7**
	CH-5	0.66	m	54.6
	CH2-6	1.30	m	28.8
	CH2-7	1.63	m	28.9
	CH-9	1.44	m	46.8
	CH2-11	1.82	d	22.6
	CH-12	5.16	bs	121.4

	CH2-16	1.63	m	24.5
	CH2-17	1.44	m	24.5
	CH-18	2.76	m	34.2
	CH-19	1.63	m	45.8
	CH2-21	1.30	m	13.8
	CH2-22	1.44	m	29.3
	CH3-23,24	0,87	t	11.4
	CH3-25,26	0.66	m	13.8
	CH3-27	1.10	m	25.4
	CH3-29	0.87	t	31.8
	CH3-30	0.87	t	22.5
**Carotenoids**	CH_2-2,2′	1.47	m	39.62
	CH_2-3,3′	1.62	m	19.27
	CH_2-4,4′	2.02	m	33.18
	CH-7,7′	6.15	d	126.68
	CH-8,8′	6.14	d	137.78
	CH-10,10′	6.14	d	130.88
	**CH-11,11′**	**6.68**	**m**	125.04
	CH-12,12′	6.35	d	137.26
	CH-14,14′	6.25	d	132.45
	CH-15,15′	6.63	m	130.02
	CH_3-16,16′,17,17′	1.03	s	29.01
	CH_3-18,18′	1.72	s	21.77
	CH_3-19,19′	1.97	s	12.81
**Phenols**	**Aromatic moieties**	**6.8-7.0**	**m**	130-140
**Aldehydes**	**CHO**	**9.76**	**bs**	204.01

[1] Metabolites identified in the ^{1}H NMR spectrum of the chloroform (C) and hexane (H) extracts of marcs; [2] In bold are evidenced the resonances chosen for metabolite quantification; [3] s: singlet, bs: broad singlet, d: doublet, t: triplet, dd: doublet of doublets, m: multiplet.

Figure S9. GC-MS qualitative analysis for CM extract

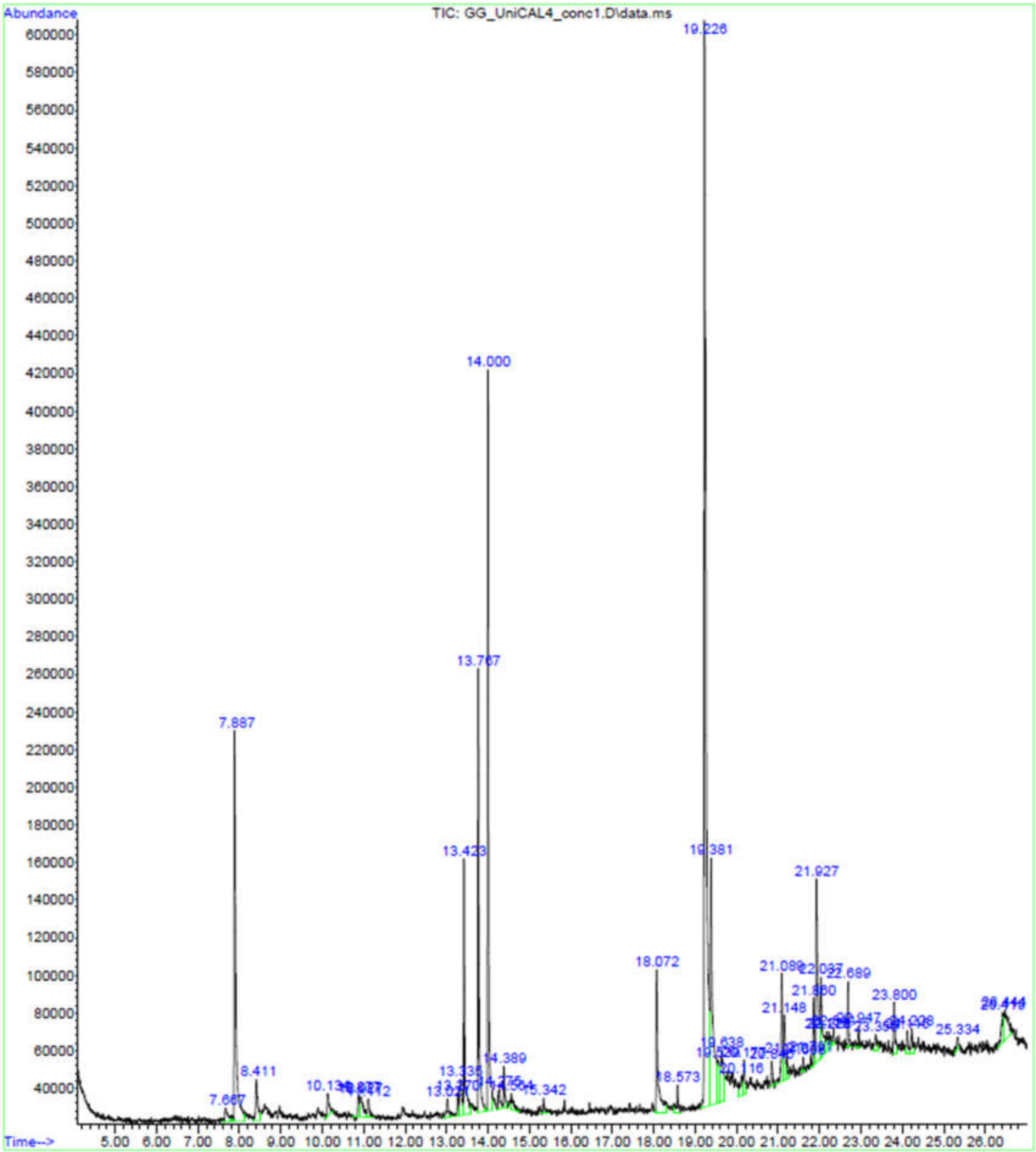

Table S2. Identification of CM peaks

RT	Area%	Library/ID	Quality
7.890	12,45	2-Heptenal, (E)-	83
13.769	9,17	2,4-Decadienal, (E,E)-	91
13.997	13,43	2,4 DECADIENAL	97
18.071	5,10	Hexadecanoic acid	96
18.570	0,86	Hexadecanoic acid	90
19.227	42,50	9,12-Octadecadienoic acid (Z,Z)-	99
19.385	11,22	Octadecanoic acid	91
19.543	2,38	9,12-Octadecadienoic acid (Z,Z)-	90
21.085	2,91	LINOLEIC ACID, BUTYL ESTER	80
**Total Identification:**		73,32%	

Figure S10. GC-MS qualitative analysis for CP extract

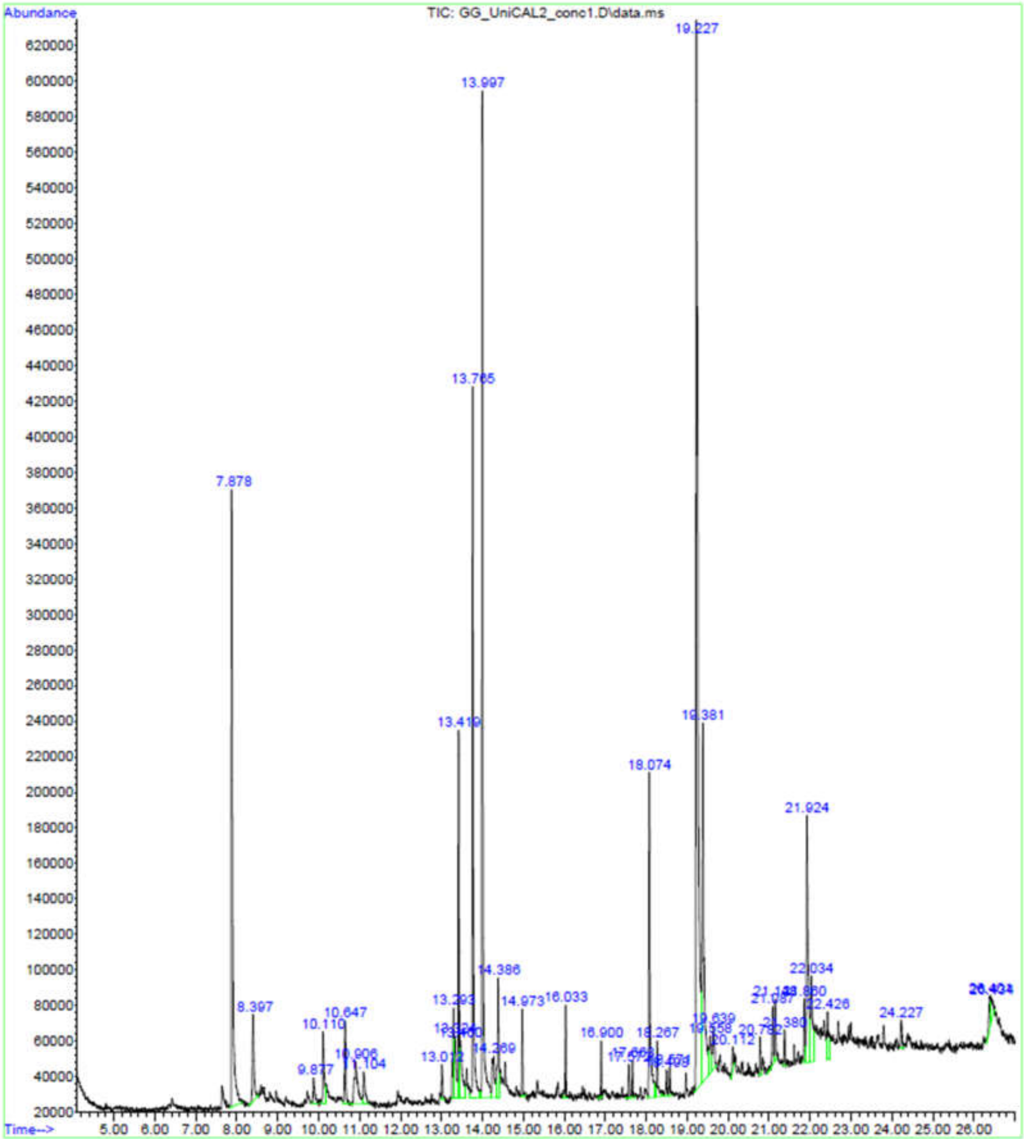

Table S3. Identification of CP peaks

RT	Area%	Library/ID	Quality
7.881	12,59	2-Heptenal, (E)-	96
8.398	2,09	1 OCTEN 3 OL	90
10.106	1,58	2 OCTENAL	90
10.650	1,58	Hexane, 1,1-diethoxy-	90
13.419	5,01	2-Decenal, (E)- (CAS) $$ trans-2-D	87
13.769	10,21	2,4-Decadienal, (E,E)-	91
13.997	15,59	2,4-Decadienal, (E,E)-	94
14.969	0,90	Cycloheptasiloxane, tetradecamethyl-	86
17.572	0,51	Phthalic acid, isobutyl nonyl ester	86
17.659	0,53	Eicosamethylcyclodecasiloxane	90
18.071	5,58	Hexadecanoic acid	98
18.264	0,98	Tetradecanoic acid, ethyl ester	90
18.571	0,50	Hexadecanoic acid	90
19.228	31,19	9,12-Octadecadienoic acid (Z,Z)-	97
19.385	9,87	Linoleic acid ethyl ester	99
19.561	1,31	9,12-Octadecadienoic acid (Z,Z)-	97
**Total Identification:**		74,26 %	

Figure S11. GC-MS qualitative analysis for HM extract

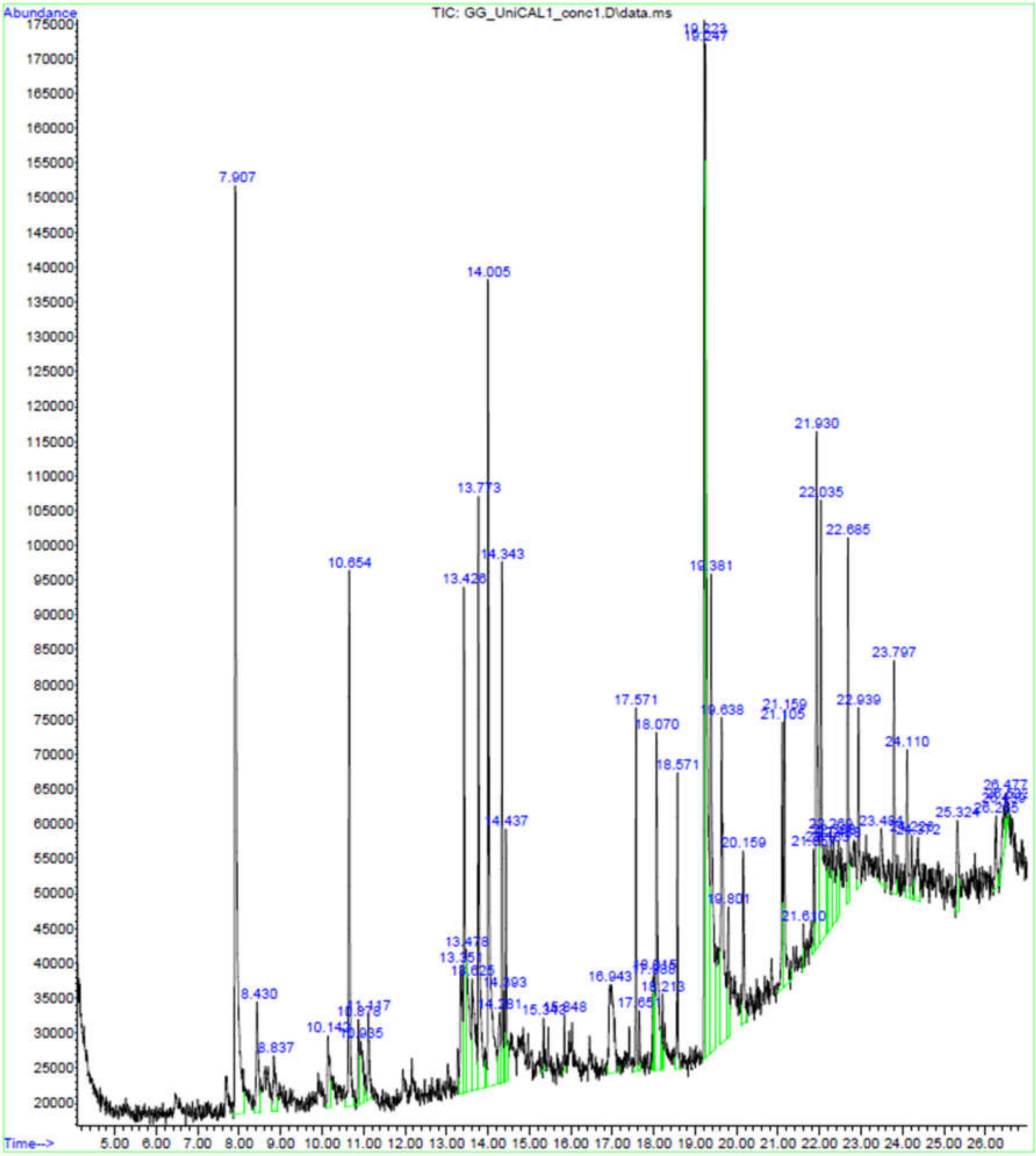

Table S4. Identification of HM peaks

RT	Area%	Library/ID	Quality
7.907	12,45	2-Heptenal, (E)-	96
10.650	5,27	Hexane, 1,1-diethoxy-	91
13.427	4,02	2-Decenal, (Z)-	86
13.769	5,95	2,4-Decadienal	91
14.005	9,76	2,4 DECADIENAL	97
16.941	3,31	1-Octanamine, N-methyl-N-octyl-	80
17.572	2,19	1,2-Benzenedicarboxylic acid, bis(2-methylpropyl) ester	90
18.071	3,96	Hexadecanoic acid	91
18.570	2,07	Hexadecanoic acid	93
19.219	6,59	9,12-Octadecadienoic acid (Z,Z)-	98
19.245	14,09	9-Octadecenoic acid (Z)-	95
19.385	7,58	Octadecanoic acid	92
19.639	5,92	9,12-Octadecadienoic acid (Z,Z)-	98
19.797	1,69	Octadecanoic acid	81
20.156	2,13	1-Octanamine, N,N-dioctyl-	86
21.103	2,50	7,12-dioxobenzo[k]fluoranthene	86
21.155	2,53	Octadecane	97
22.031	5,57	Eicosane	95
22.689	2,41	13-Hexacosyne	80
**Total Identification:**		70,73 %	

Figure S12. GC-MS qualitative analyses for HP extract

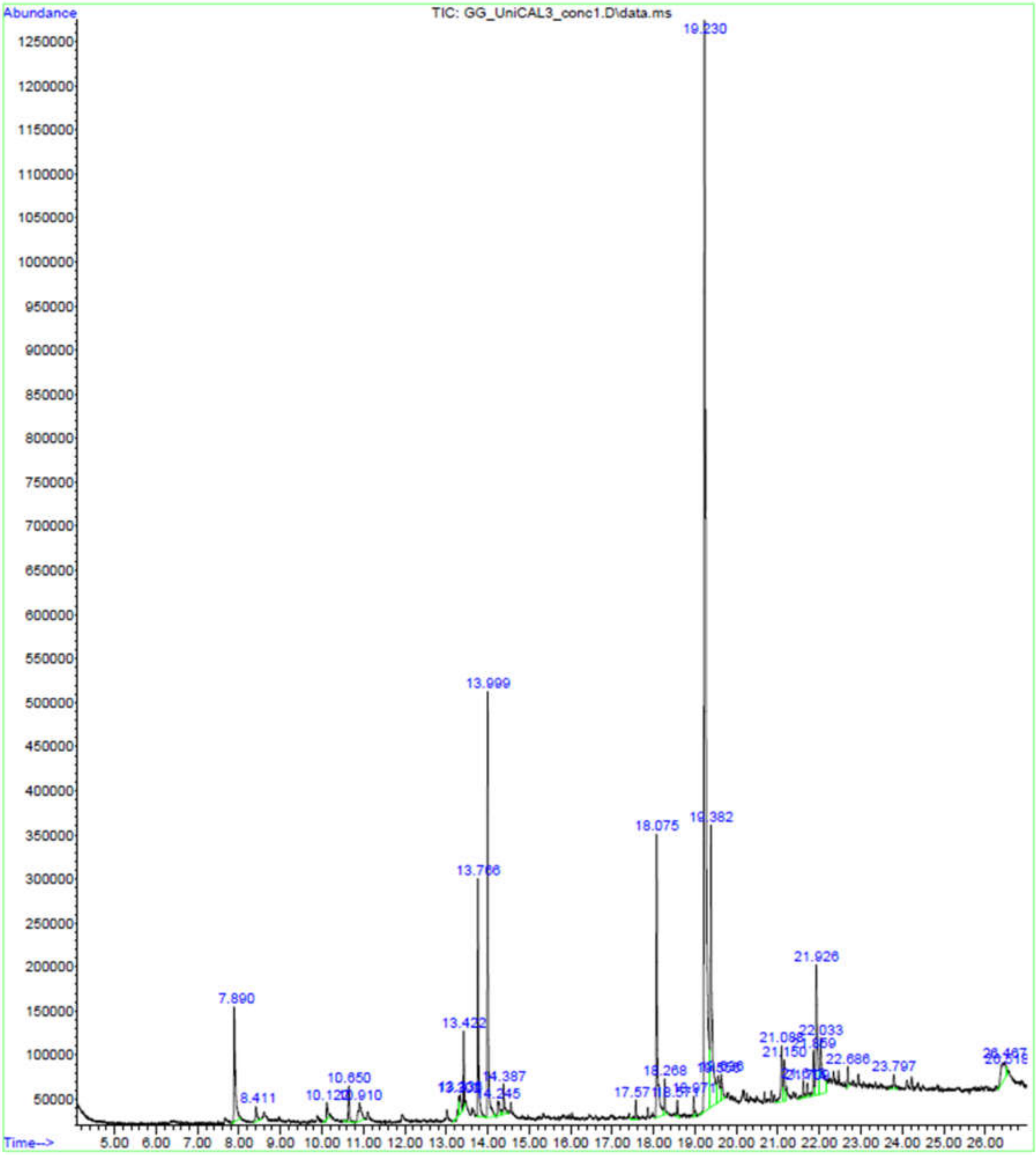

Table S5. Identification of HP peaks

RT	Area%	Library/ID	Quality
7.890	4,53	2-Heptenal, (E)-	90
10.124	0,59	2-Octenal, (E)-	90
10.650	1,18	Hexane, 1,1-diethoxy-	91
13.418	1,48	2-Decenal, (E)-	80
13.769	5,83	2,4-Decadienal, (E,E)-	91
13.997	10,37	2,4 DECADIENAL	97
14.382	0,93	E-2-dodecenal	80
17.572	0,53	1,2-Benzenedicarboxylic acid, bis(2-methylpropyl) ester	86
18.071	8,03	Hexadecanoic acid	99
18.264	0,89	Hexadecanoic acid, ethyl ester	99
18.570	0,52	Hexadecanoic acid	93
19.228	49,60	9,12-Octadecadienoic acid (Z,Z)-	99
19.385	12,30	9,12-Octadecadienoic acid, ethyl ester	97
19.552	1,54	9,12-Octadecadienoic acid (Z,Z)-	93
19.639	1,20	9,12-Octadecadienoic acid (Z,Z)-	86
21.611	0,48	Eicosane	93
**Total Identification:**		81,07%	

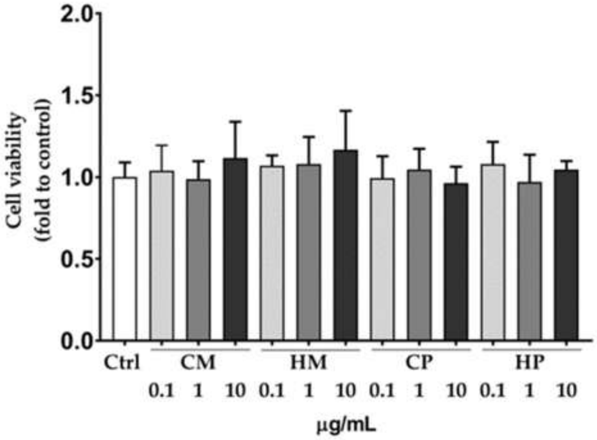

Figure S13. HaCaT cell viability assay
